# First Draft Genome Sequence of Wheat Spot Blotch Pathogen *Bipolaris sorokiniana* BS_112 from India, Obtained Using Hybrid Assembly

**DOI:** 10.1128/MRA.00308-19

**Published:** 2019-09-19

**Authors:** Rashmi Aggarwal, Sapna Sharma, Kartar Singh, Malkhan Singh Gurjar, Mahender Singh Saharan, Sangeeta Gupta, Bishnu Maya Bashyal, Kishor Gaikwad

**Affiliations:** a Fungal Molecular Biology Laboratory, Division of Plant Pathology, ICAR-Indian Agricultural Research Institute, New Delhi, India; b ICAR-National Research Centre on Plant Biotechnology, New Delhi, India; Vanderbilt University

## Abstract

Bipolaris sorokiniana is a devastating fungal pathogen causing spot blotch of wheat. We report here the first draft genome of *Bipolaris sorokiniana* strain BS_112 from India using sequence reads from the Ion Torrent, Illumina HiSeq, and Nanopore platforms. The genome size was estimated at 35.64 Mb with an average G+C content of 50.20%.

## ANNOUNCEMENT

Spot blotch caused by Bipolaris sorokiniana (phylum Ascomycota, class Dothideomycetes) is one of the most devastating diseases of wheat in the warmer areas of the world (1). The disease is distributed widely across the wheat-growing areas of the world, including Africa, South America, Australia, Canada, Asia, and particularly the Indian subcontinent under the prevalent warm and humid conditions. Yield losses of between 20 and 80% have been reported and may lead to complete devastation under the most severe conditions (2). The pathogen is hemibiotrophic, initially biotrophic and turning to a necrotrophic phase at later stages (3). PCR-based DNA fingerprinting grouped *B. sorokiniana* isolates according to their geographic locations in India (1). A sequence-characterized amplified region (SCAR) marker has been developed for detection of *B. sorokiniana* (4), but little is known about the molecular basis of pathogenesis in *B. sorokiniana* due to a paucity of resources for molecular genetics. Therefore, a draft genome sequence of *B. sorokiniana* was generated, assembled, and analyzed.

High-quality genomic DNA was extracted from monoconidial culture of a highly virulent strain of *B*. *sorokiniana*, BS_112 (GenBank accession number KU201275), grown at 26°C with constant shaking (150 rpm) in potato dextrose broth (HiMedia, India) for 3 to 4 days using a Qiagen DNeasy minikit. Then, the quality and quantity were assessed using a spectrophotometer, Qubit fluorometer, and Agarose gel electrophoresis. This pathogen was sequenced using three platforms, *viz.*, Illumina HiSeq, Ion Torrent, and Nanopore, following the manufacturers’ instructions, including library preparation and sequencing. The TruSeq DNA PCR-free kit was used for Illumina library preparation with an insert size of 350 bp and a read size of 150 bp from each end, and a total of 36,915,848 paired-end reads were obtained. In the Ion Torrent library preparation, the fungal DNA was enzymatically sheared into suitably sized fragments using the Ion Xpress Plus fragment library kit. The barcode was applied, and the adapter-ligated library was size selected for a target peak of 330 bp using E-Gel Size Select 2% agarose gel (Invitrogen Corporation, USA). Finally, the adapter-ligated and size-selected library was amplified using the Ion Plus fragment library kit (Life Technologies [LT], USA), and the run was performed on an Ion PGM system version 1. A total of 845,589 reads were obtained using the Ion Torrent platform. The library preparation for the Oxford Nanopore platform was performed using a ligation sequencing kit (SQK-LSK108) from Oxford Nanopore Technologies according to the instructions provided with the kit, and a total of 560,150 reads were generated. The Illumina reads were preprocessed using Trim Galore version 0.4.0 (5); Nanopore Fast5 data were base called using Albacore version 2.3.1 (6), and for the Ion Torrent reads, the bases with a quality (Q) score of ≥20 were considered for assembly. All three types of sequencing reads were used for the hybrid genome assembly using the MaSuRCA tool version 3.2.2 (7). Further, the contigs were processed to scaffolds using pyScaf version 3 (https://pypi.org/search/?q=PyScaf+version+3). The generated whole-genome size is 35.64 Mb with a G+C content of 50.2%, 235 scaffolds, and an *N*_50_ value of 1,654,800 bp. BUSCO provided quantitative measures for the assessment of the genome assembly using ortholog groups, and the evaluation was performed using fungal data sets. The BUSCO evaluation of completeness of the *B. sorokiniana* genome sequence assembly predicted that it was complete to about 97.6%. The gene prediction was performed using the AUGUSTUS tool version 2.2.5 (8) with the reference *Bipolaris sorokiniana* ND90Pr (GenBank accession number AEIN00000000). A total of 10,460 genes were predicted with an average gene density of 250 to 300 genes/Mb, which covers around 98% of predicted genes. The average gene length was 435 to 545 bp, the maximum gene length was 8,506 bp, and the minimum gene length was 50 bp. Gene ontology (GO) annotations of the genes were determined using Blast2GO version 2 (9). Out of 10,460 annotated genes, 1,024 (3,491), 493 (4,165), and 1,274 (7,784) genes (hits) were for biological processes, cellular components, and molecular functions, respectively. The *B. sorokiniana* genome was interrogated with the Pathogen Host Interactions (PHI) database version 4.3 (10), and in total, 3,627 genes were found to be homologous to proteins in the PHI database with an E value of ≤1e-05. Single-nucleotide polymorphism (SNP) prediction was processed using the SnpEff tool version 3.3h (11) with the Illumina HiSeq data and identified 93,122 variants containing 88,672 SNPs and 4,450 indels. Further, simple sequence repeat (SSR) identification using the MISA tool version 1 (https://webblast.ipk-gatersleben.de/misa/) showed 5,996 SSRs, and 146 of the 235 SSR-containing sequences were examined. The genome sequence of *Bipolaris sorokiniana* ND90Pr was used as reference for the analysis of SNPs and SSRs.

### Data availability.

This whole-genome sequencing project has been deposited in DDBJ/ENA/GenBank under the accession number RCTM00000000, BioProject number PRJNA489551, and BioSample identification (ID) SAMN09901300. The version described in this paper is the first version (GenBank accession number RCTM01000000). The internal transcribed spacer (ITS) sequence was deposited at NCBI with the accession number KU201275. The raw sequencing data of all the three platforms were deposited in the DDBJ SRA database under the accession numbers SRX6673825 (Ion Torrent), SRX6673824 (Illumina HiSeq), and SRX6100476 (Oxford Nanopore).
